# Bayesian modeling of Dynamic Contrast Enhanced MRI data in cerebral glioma patients improves the diagnostic quality of hemodynamic parameter maps

**DOI:** 10.1371/journal.pone.0202906

**Published:** 2018-09-26

**Authors:** Anna Tietze, Anne Nielsen, Irene Klærke Mikkelsen, Mikkel Bo Hansen, Annette Obel, Leif Østergaard, Kim Mouridsen

**Affiliations:** 1 Charité - Universitätsmedizin Berlin, corporate member of Freie Universität Berlin, Humboldt-Universität zu Berlin, and Berlin Institute of Health, Germany; 2 Center of Functionally Integrative Neuroscience, Clinical Institute, Aarhus University, Aarhus, Denmark; 3 Dept. of Neuroradiology, Aarhus University Hospital, Aarhus, Denmark; Beijing University of Technology, CHINA

## Abstract

**Purpose:**

The purpose of this work is to investigate if the curve-fitting algorithm in Dynamic Contrast Enhanced (DCE) MRI experiments influences the diagnostic quality of calculated parameter maps.

**Material and methods:**

We compared the Levenberg-Marquardt (LM) and a Bayesian method (BM) in DCE data of 42 glioma patients, using two compartmental models (extended Toft’s and 2-compartment-exchange model). Logistic regression and an ordinal linear mixed model were used to investigate if the image quality differed between the curve-fitting algorithms and to quantify if image quality was affected for different parameters and algorithms. The diagnostic performance to discriminate between high-grade and low-grade gliomas was compared by applying a Wilcoxon signed-rank test (statistical significance p>0.05). Two neuroradiologists assessed different qualitative imaging features.

**Results:**

Parameter maps based on BM, particularly those describing the blood-brain barrier, were superior those based on LM. The image quality was found to be significantly improved (p<0.001) for BM when assessed through independent clinical scores. In addition, given a set of clinical scores, the generating algorithm could be predicted with high accuracy (area under the receiver operating characteristic curve between 0.91 and 1). Using linear mixed models, image quality was found to be improved when applying the 2-compartment-exchange model compared to the extended Toft’s model, regardless of the underlying fitting algorithm. Tumor grades were only differentiated reliably on plasma volume maps when applying BM. The curve-fitting algorithm had, however, no influence on grading when using parameter maps describing the blood-brain barrier.

**Conclusion:**

The Bayesian method has the potential to increase the diagnostic reliability of Dynamic Contrast Enhanced parameter maps in brain tumors. In our data, images based on the 2-compartment-exchange model were superior to those based on the extended Toft’s model.

## Introduction

Dynamic Contrast Enhanced (DCE) MRI enables the estimation of hemodynamic parameters in tissue and is used to investigate the microvasculature and blood-brain-barrier permeability in a variety of brain pathologies [1–4]. Clinical trials indicate the usefulness of this technique for evaluating for example the treatment response in brain tumor patients [4–6]. DCE has, moreover, the potential to become an important component of routine clinical management of tumor patients, as microvascular structure and function are altered during the course of radio-chemotherapy [3, 7]. DCE is in many respects superior to the clinically more prevalent Dynamic Susceptibility Contrast (DSC) MRI method, because it appears more reliable in the presence of contrast agent leakage across a corrupted blood-brain barrier [8], provides quantitative measurements, and as DCE is a T1-weighted technique, it is less affected by susceptibility artifacts. This allows the evaluation of brain regions in the vicinity of air-containing structures as the posterior fossa or the temporal lobes, but also around hemorrhages, or adjacent to surgical devices as coils, clips, catheters, sensors etc. In addition, DCE is the option for performing gadolinium-based perfusion imaging outside the brain.

However, insufficient imaging protocols, poor image quality, and unreliable parameter estimations have so far prevented the more widespread DCE implementation of DCE in neuroimaging so far. Recent improvements in scanner hard- and software have substantially minimized acquisition issues, but data post-processing remains a challenge. The clinician is often presented with noisy and unreliable parameter maps, which keeps hampering the acceptance of DCE within the clinical community and calls for an improved approach to data analysis.

DCE data are typically analyzed through employment of compartmental models (see ref. [9] for a review). A popular model and one that has been used extensively in clinical studies is the three-parameter extended Toft’s model (ETM) [10], which enables the measurement of the vascular-to-extravascular leakage parameter K_trans_, the intra-vascular plasma volume v_p_, and the extravascular interstitial volume v_e_. Alternatively, the so-called 2-compartment-exchange model (2CXM) [11] is employed, which enables the separation of K_trans_ into the permeability rate K_1_ and the plasma flow F_p_. However, parameter estimation depends more critically on temporal sampling and is more affected by data noise [12], which requires a robust fitting algorithm.

The standard approach to estimate the parameters of the ETM or the 2CXM models is to fit one of these models to some observed data using the so-called Levenberg-Marquardt (LM) method [13, 14]. Here, the sum of the squared differences between model and observed data points is minimized in order to find an optimal solution. However, due to the complexity of the problem, this simple approach may converge to a non-optimal local minimum in the cost function space, with the resulting parameters bearing no or only limited physiological meaning. This problem might be identified by spurious voxels in the resulting hemodynamic parameter maps and can, if present to a substantial degree, lead to considerable image degradation.

An alternative algorithm for estimating the model parameters is a Bayesian method (BM). The BM is a probabilistic approach based on the assumption that the model parameters follow a normal distribution. The mean and covariance of this distribution are determined (prior distribution) and afterwards used to infer the posterior distribution of the measured variables by maximizing the log-likelihood function. This has previously been observed to be more robust with respect to image noise and provides substantial improvements in tissue differentiation, while at the same time being capable of estimating perfusion indices independently across a wide range of values [15–18]. Bayesian models have also been employed in DCE models before and appear to yield consistent and accurate results [19, 20].

While our phantom studies and initial clinical examples published in the same issue of this journal indicate that BM allows estimation of compartmental parameters, which are more robust against experimental noise (“Robust estimation of hemo-dynamic parameters in traditional DCE-MRI models”; submitted to PLoS One, PONE-D-18-01462), the immediate clinical utility has not been examined. Here we compare the diagnostic quality of hemodynamic maps based on the conventional LM to those resulting from BM. We evaluate the impact of both curve-fitting algorithms on two compartmental models (ETM and 2CXM), calculated from DCE data acquired in 42 untreated cerebral glioma patients. The primary focus is on the clinical usefulness, i.e. if the calculated maps enabled better assessment of tumor heterogeneity, to distinguish between pathological and normal appearing tissue, or better separation of tumor vasculature from normal vessels. The aim of our study is hereby to assess image quality and the extent of spurious voxels and image artifacts, potentially distorting quantitative analyses.

## Materials and methods

### Patients

This study was approved by the Danish Committee on Health Research Ethics. All included patients provided informed written consent to be part of this study and to have their data used in research. MRI data were pseudo-anonymized after collecting. The study population consisted of 42 untreated glioma patients [23 glioblastoma, 7 astrocytoma grade III, 2 oligodendroglioma grade III, 10 astrocytoma grade II; all of them diagnosed by either biopsy or resection).

### Imaging

MRI data were acquired on an Achieva 3.0 T Philips system (Philips Healthcare, Best, Netherland) with a standard eight-element head coil. DCE imaging was performed using a turbo-FLASH sequence (TR/TE = 3.5/1.57 ms; flip angle = 25˚; voxel size = 1.7x1.7x5 mm^3^; FOV = 220 x 220 x 75 mm^3^; dynamic scan time = 2s; dynamic scan duration = 5 min.) before, during, and after the administration of 0.05 mmol/kg gadobutrol at 2.5 ml/s, flushed with 30 ml saline at 2.5 ml/s. Baseline T1 values were measured and corrected for B1 field inhomogeneity as described in [21].

### Image analysis

Post-processing was done using in-house developed modules run in SPM8 (Statistical Parametric Mapping, Wellcome Trust Centre for NeuroImaging, Inst. of Neurology, University College London, UK) and MatLab (Mathworks, Natick, MA, USA). B1 field-corrected T1 maps were estimated, and the DCE concentration-to-time curves were obtained as described previously [21]. The ETM and 2CXM were fitted to the concentration-to-time curves using the two curve-fitting algorithms. In LM, a penalty to the sum-of-squares cost function was added if parameters were out of a physiological range (F_p_: 0–500 ml/100g/min; Ve: 0–1 ml/g; v_p_: 0–1 ml/g; K1: 0–100 ml/100g/min). This was carried out by adding the arbitrary number of 100,000 to the sum-of-the squares, such as to discourage the algorithm from pursuing this route. For BM, the expectation-maximization optimization method in a Bayesian framework was used as described in refs. [15, 16]. To avoid negative values parameter estimation was performed on log-transformed parameters, with no upper bounds on the parameters in BM. The starting guess (LM) and prior mean (BM) used to initialize the curve fitting procedures was: K_1_ and v_p_ from the Patlak’s model [22], F_p_ and K_trans_ using a standard singular value decomposition [23], and V_e_ as the area under the curve (AUC) of the tissue concentration curve (from 60s after baseline to 300s). For BM, it is also necessary to specify a prior parameter covariance matrix. Similar to ref. [24], we chose the prior covariance as a diagonal matrix with entries, which, through simulation studies, were found to allow an independent and robust estimation of DCE model parameters over wide ranges of values. Specifically, for ETM we used a prior covariance matrix with diagonal elements (0.1, 10, 0.1, 10) for the parameters (F_p_, F_p_/v_e_, v_p_, delay), while for 2CXM the prior covariance matrix (0.1, 1, 10, 1, 10) for the parameters (F_p_, v_p_, F_e_, v_e_, delay) was applied (for details the reader is referred to “Robust estimation of hemo-dynamic parameters in traditional DCE-MRI models”; submitted to PLoS One, PONE-D-18-01462). Data were corrected for internal motion if necessary. The arterial input function was obtained semi-automatically using MatLab from either the anterior or middle cerebral artery on the unaffected side. To improve local signal-to-noise ratios, spatial 2D smoothing was performed with a 3 x 3 voxel uniform filter on the concentration maps prior to perfusion calculation. While this may blur edges between different tissue compartments, such effects were outweighed by the benefit in increased hemodynamic parameter map quality. We also note that Schmid et al. [19] have put forward a Gaussian Markov random field solution to incorporate local spatial information in the prior distribution, which may alleviate such phenomena.

### Qualitative image analysis

**Fig 1:**
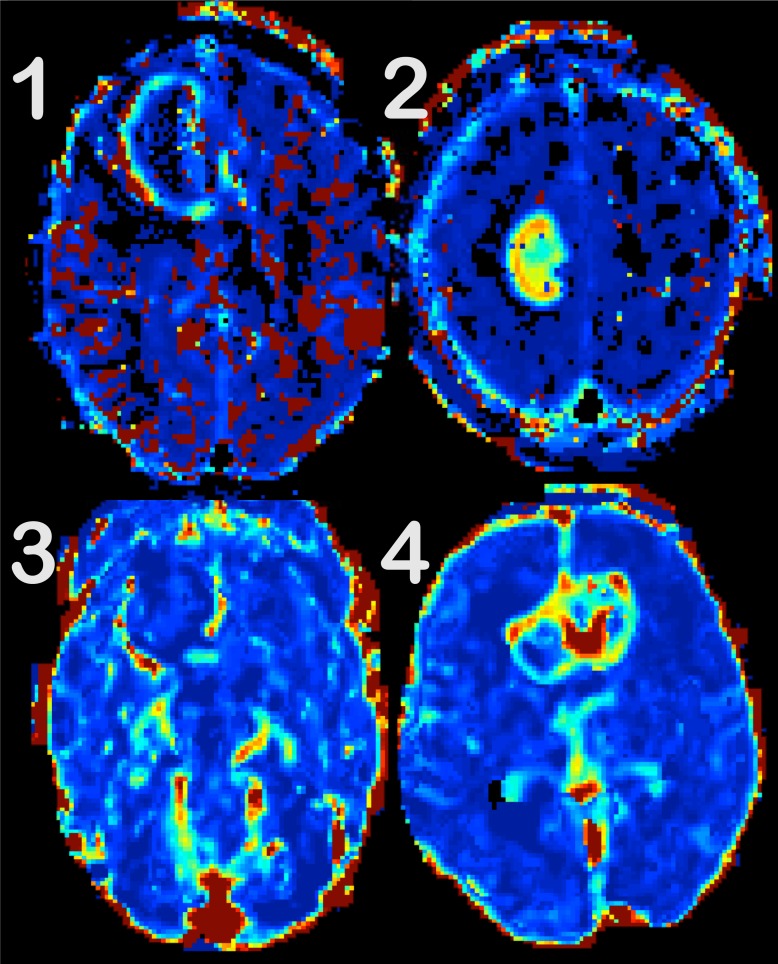
Examples of the 4-step-scale, used for quality assessment. Tumor-to background discrimination is evaluated for V_e_ maps. (1) is not interpretable, as spurious voxels (in red and black) partly obscure the tumor. In (2), the tumor is discernible, but still affected by artifacts both in and around the lesion. The image quality is good in (3), but the tumor is difficult to spot due to the surrounding structures and artifacts. The tumor-to-background discrimination is excellent in (4).

The above analysis resulted in 7 different parametric perfusion maps (*K*_1_, *v*_p_, *F*_p_, *v*_e_ from the 2CXM; *K*_trans_, *v*_p_, *v*_e_ from the ETM) for each patient and each fitting algorithm (LM and BM). These maps were presented for two board certified neuroradiologists (reader 1: 6 years of experience in neuroradiology, reader 2: 15 years of experience), ordered by hemodynamic parameters, but in a random order with respect to the underlying fitting algorithm. Three image features were scored: (i) tumor-to-background discrimination (t2b), (ii) tumor-to-‘surrounding normal vessels’ discrimination (t2v), and (iii) overall impression (oai). A 4-step-scale was used with 1 = not interpretable due to severe image degradation, 2 = considerable image artifacts limiting interpretation, 3 = good quality, and 4 = excellent quality. For examples, see Fig 1.

### Statistical analysis

Statistical analysis was performed using R (Boston, MA, USA).

For an overall illustration of clinical assessment scores, we calculated and plotted the average scores for image features t2b, t2v and oai for the 7 parameter maps (3 in the ETM and 4 in the 2CXM) for both raters and both fitting algorithms. In addition, we looked at the average scores for high-grade and low-grade gliomas separately, in order to assess if tumor grade and, thus, the extent of contrast agent leakage or neo-angiogenesis might influence image quality indirectly.

Two complementary statistical approaches were used to quantify the differences in image quality between the two algorithms. First, logistic regression was used to differentiate between the two fitting algorithms. This was done in order to investigate whether the image quality, as quantified by the scores, was sufficiently different to make this grouping possible. With 168 samples (42 patients * 2 raters * 2 fitting algorithms) we fitted a model with the type of fitting algorithm as response and the quality scores (7 parameters * 3 image features [t2b, t2v, oai]) as predictors. To protect against overfitting the data were divided into a training set (120 samples) and a test set (48 samples). The parameters of the logistic regression were estimated with the training set. The resulting model was subsequently applied to the test data, and the accuracy to determine whether the samples in the test set were based on LM or BM was evaluated. The training and estimation procedure was repeated on 1000 randomly sampled training and test sets. A penalty was used in the logistic regression to enable automatic variable selection [25]. The selected variables reflect which combination of the 7 parameter maps and 3 image features were most informative in distinguishing between the two fitting algorithms for each repetition. The ability to differentiate between the two fitting algorithms based on clinical image quality was quantified by the area under the receiver operating characteristic (ROC) curve (AUC).

Second, we estimated the extent to which fitting algorithm, parameter, and image features affected clinical scores. We used an ordinal linear mixed model [26] with score as dependent variable (response) and algorithm, parameter, image feature, and rater as independent explanatory variables. Random effects were added for patients and raters to account for the correlation between repeated measures. The ordinal linear mixed model was fitted using an expectation-maximization algorithm (EM) [26]. The EM approach enabled the calculation of p-values and thereby testing of the significance of the individual explanatory variables.

Finally, we evaluated if the two fitting algorithms had an impact on the diagnostic performance at all by using by K_trans_/K_1_ and v_p_ parameter maps, calculated by 2CTX and ETM, respectively, to discriminate between high-grade and low-grade gliomas. The mean K_trans_, K_1_, and v_p_ were determined in the contrast enhancing part of the tumor or in the T2FLAIR hyperintense part for non-enhancing lesions and normalized by the mean value in non-affected white matter. We applied a Wilcoxon signed-rank test and regarded a p<0.05 as statistically significant. Furthermore, we calculated the ROC curves and AUCs for K_trans_, K_1_, and v_p_ and tumor grades.

## Results

### Qualitative evaluation of patient data

**Fig 2:**
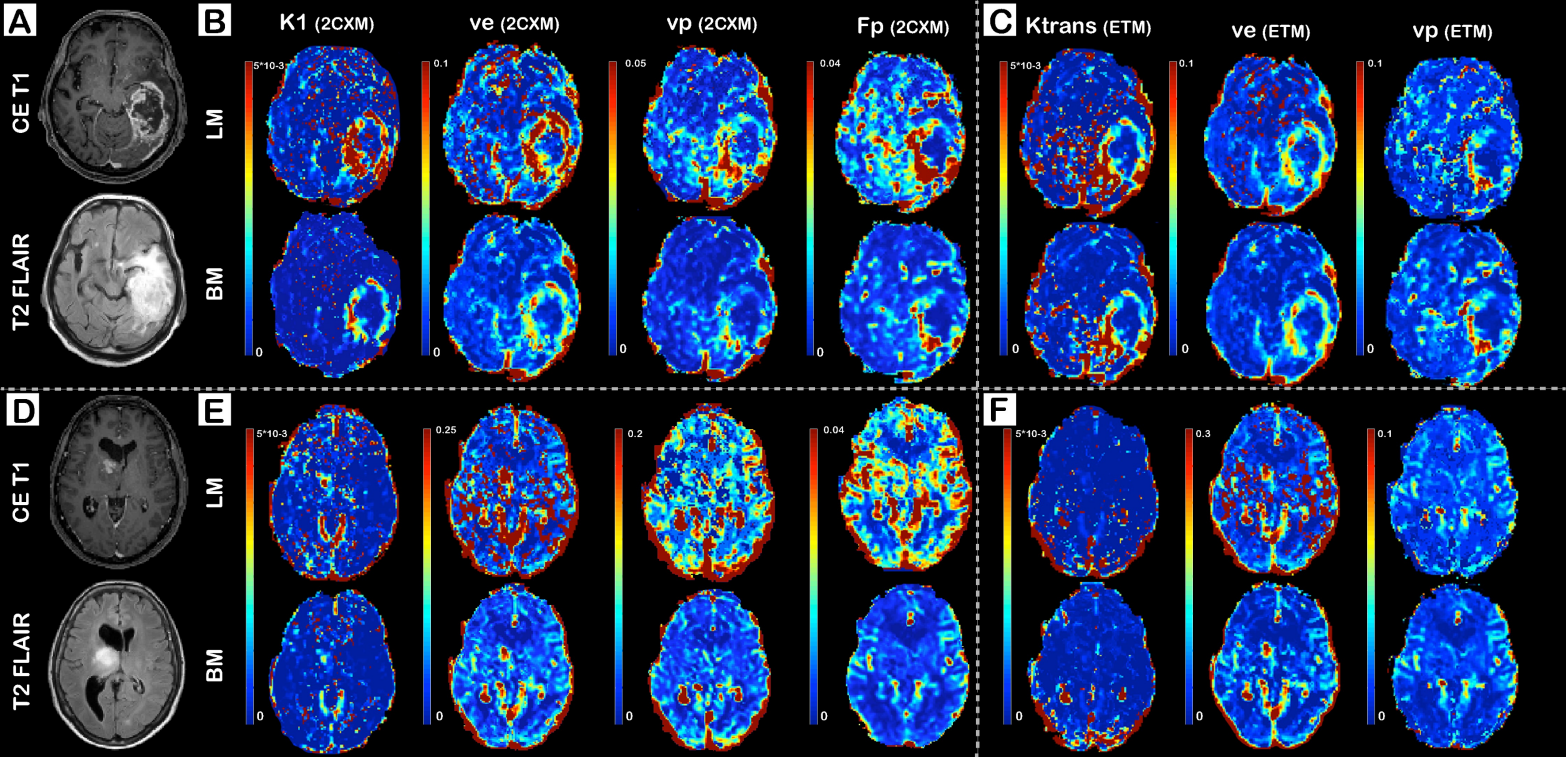
Representative K_1_ (permeability rate), K_trans_ (leakage-flow), V_e_ (interstitial volume), v_p_ (plasma volume), and F_p_ (plasma flow) images of a patient with glioblastoma multiforme (A-C) and astrocytoma grade II (D-F). Dynamic Contrast Enhanced MRI data are fitted to the 2-compartment-exchange model (2CXM; B and E) and the extended Toft’s model (ETM; C and F) using either the Levenberg-Marquardt (LM) method or Bayesian modeling (BM). Conventional images (contrast enhanced T1, CE T1, and T2 FLAIR) are demonstrated in (A) and (D).

Representative images for a high-grade glioma (glioblastoma multiforme) and a low-grade glioma (astrocytoma grade II) are shown in Fig 2A–2F. Fig 2B and 2E demonstrates K_1_, v_p_, F_p_, and V_e_ maps based on 2CXM, whereas Fig 2C and 2F displays K_trans_, v_p_, _e_ from the ETM. Data are fitted with the LM method in the upper row and with the BM approach in the bottom row of the subfigures B, C, E, and F. Sensitivity to image artifacts is particularly apparent on LM-based maps, but only to minor degree on the BM-based K_trans_ maps. The remaining BM maps are largely unaffected by artifacts. Spurious hyperintensity voxels disguise partly the tumor or the transition zone between tumor and normal appearing tissue, which impedes the t2b discrimination, the assessment of tumor heterogeneity, and the reliable identification of small satellite lesions. The difference between LM- and BM-fitted parameter maps is most apparent on v_e_ images and to a minor degree on K_1_, and K_trans_ maps.

**Fig 3:**
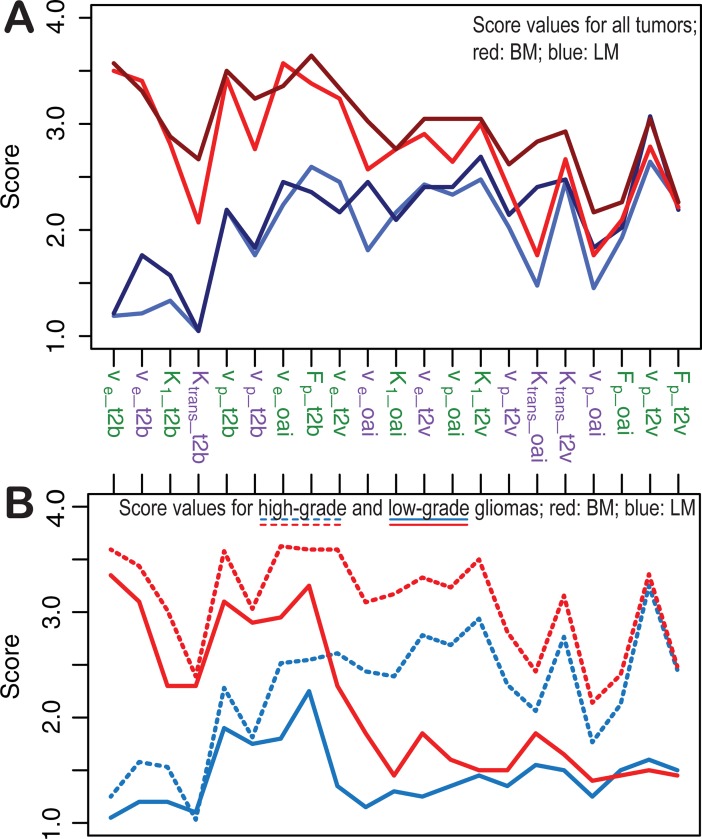
Observed mean score values in all tumors (A): Images based on the Bayesian model are scored higher (red colors with light red for rater 1 and dark red for rater 2) than those based on the Levenberg-Marquardt method (blue colors with light blue for rater 1 and dark blue for rater 2) for most parameter maps and imaging features. A sub-analysis (B; averaged for both rater) for high-grade (dashed lines) and low-grade gliomas (solid lines) suggests that this is most pronounced for tumor-to-background (t2b) discrimination. Green labels indicate parameters from the 2-compartment-exchange (2CXM), purple labels those from the extended Toft’s model (ETM). t2v: tumor-to-‘surrounding normal vessels’ discrimination; oai: overall impression. V_e_: interstitial volume; K_1_: permeability rate; v_p_: plasma volume; K_trans_: leakage-flow parameter; F_p_: plasma flow.

In Fig 3A, we show the average scores for the different parameters maps. Scores of BM-based maps are plotted in red, those of LM-based maps in blue (rater 1 in light, rater 2 in dark color). It is observed that the raters consistently rank BM-based maps higher than LM-based maps, regardless of model (green: 2CXM; purple: ETM), parameter, and image feature (t2b, t2v, oai). The difference is most profound in t2b assessment, illustrating the excellent tumor demarcation when using BM. In Fig 3B, the scores (here averaged for both raters) are plotted separately for high-grade and low-grade tumors (high-grade with dashed, low-grade with solid lines; BM in red and LM in blue). The figure suggests that the algorithm influences primarily the t2b, whereas the tumor grade dominates when assessing t2v and oai.

The logistic regression shows that the two fitting algorithms can be differentiated by just using scores. The average AUC is 0.98 (confidence interval ± 0.018) with the lowest being 0.91, and an AUC of 1 was reached in 26% of repetitions. The automatic variable selection yields that the quality of t2b on v_e_ maps (2CXM) is sufficient in 58% of cases to identify the fitting algorithm (lowest AUC 0.91; mean AUC 0.91, confidence interval ± 0.019). In the remaining cases, classification was made with K_1_ (2CXM), K_trans_, and v_e_ (ETM) maps, illustrating that the image quality of leakage maps can be improved by using BM.

**Fig 4:**
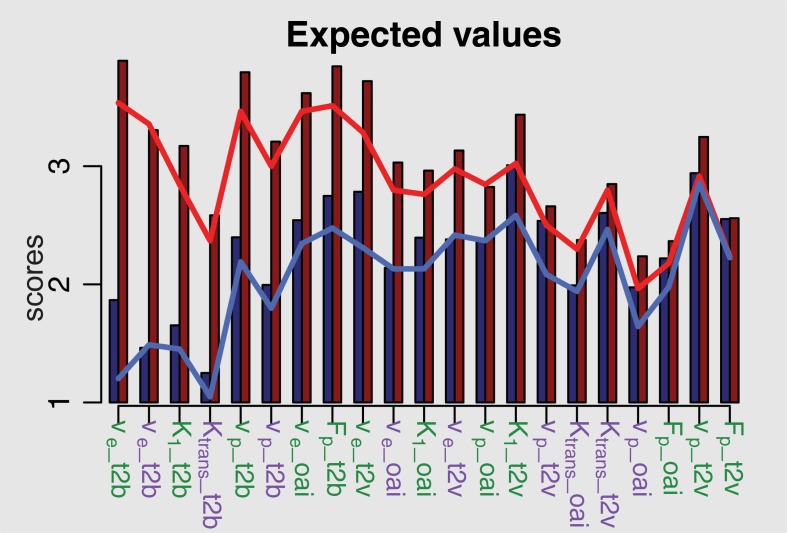
Results of the ordinal mixed model, estimating the predicted scores of image quality, which is generally predicted to be higher using Bayesian modeling (BM, red columns) compared to the Levenberg-Marquardt method (LM, blue columns). The blue line represents the mean observed values of the LM-based scores, whereas the red shows BM-based mean values. V_e_: interstitial volume; K_1_: permeability rate; v_p_: plasma volume; K_trans_: leakage-flow parameter; F_p_: plasma flow. Green labels indicate parameters from the 2-compartment-exchange (2CXM), purple labels those from the extended Toft’s model (ETM).

In Fig 4, the results of the ordinal linear mixed model are presented with predicted values as bars and observed values as lines (blue are LM-based and red are BM-based scores). The figure shows good agreement between the observed and predicted values with just slight overestimation. The scores are generally predicted to be higher on BM-based (red bars) than on LM-based maps (blue bars). The results of the ordinal linear mixed model are summarized in Table 1. In order to assess if the algorithm is critical for scoring, we removed it as an explanatory variable and found it to be significant with p<0.001. Moreover, Fig 4 shows that BM yields particularly high scores when assessing t2b. This is emphasized by the large positive coefficient of BM (1.518) in Table 1, whereas t2v and oai were less affected by the type of algorithm (illustrated by the negative interactions t2v:BM and oai:BM). This is consistent with the findings from Fig 3. Changing from 2CXM to ETM reduced the expected scores for all image features and parameters, regardless of the algorithm type (as the coefficient of ETM is negative).

**Table 1:** Coefficients, standard error, t- and p-values from the fitted mixed linear model. Baseline parameters are represented with coefficient 0.

	Value	Std.Error	t-value	p-value
Intercept	1.250	0.100	12.567	0.000
K_1_ or K_trans_	0.000			
F_p_	1.095	0.083	13.238	0.001
V_e_	0.213	0.066	3.244	0.000
V_p_	0.745	0.066	11.331	0.000
t2b	0.000			
t2v	1.334	0.065	20.657	0.000
oai	0.830	0.065	12.847	0.000
LM	0.000			
BM	1.518	0.065	23.661	0.000
rater 1	0			
rater 2	0.194	0.025	7.681	0.000
2CXM	0.000			
ETM	-0.270	0.038	-7.108	0.000
BM:ETM	-0.184	0.054	-3.425	0.001
F_p_:BM	-0.421	0.085	-4.963	0.000
V_e_:BM	0.508	0.066	7.725	0.000
V_p_:BM	-0.121	0.066	-1.840	0.066
F_p_:t2v	-1.551	0.099	-15.722	0.000
V_e_:t2v	-0.438	0.081	-5.433	0.000
V_p_:t2v	-0.813	0.081	-10.090	0.000
F_p_:oai	-1.271	0.099	12.885	0.000
V_e_:oai	-0.06	0.081	-0.813	0.416
V_p_:oai	-0.762	0.081	-9.461	0.000
t2v:BM	-1.090	0.061	-17.908	0.000
oai:BM	-0.951	0.061	-15.617	0.000
ETM:rater 2	0.267	0.050	5.352	0.000
2tv:rater 2	-0.043	0.061	-0.703	0.482
oai:rater 2	0.175	0.061	2.894	0.004

Table 1 shows a positive coefficient for rater 2. This is consistent with Fig 3, where the dark lines representing the scores given by rater 2 are above the lines representing the scores giving by rater 1. The positive coefficient of the interaction ETM:rater 2 means that this difference is most distinct, when ETM is used as model.

The diagnostic performance to discriminate high-grade from low-grade gliomas is higher for the BM-based approach when using v_p_ maps (for both 2CXM and ETM with p<0.001 and AUCs of 0.97 and 0.87; for LM p>0.05 for both models, AUCs of 0.69 and 0.62) and equal for K_1_ and K_trans_ maps (K_1_ with p>0.05, AUCs of 0.57 and 0.56, and K_trans_ with p<0.001, AUCs of 0.92 and 0.92).

## Discussion

We have shown that the diagnostic quality of most of our BM-based parameter maps is superior to those fitted with the widely used LM-based approach, which was here even adjusted to avoid erroneously high parameter estimates. Especially the leakage-related parameters K_1_, K_trans_, and v_e_ appear to benefit from using the Bayesian parameter estimation. Our qualitative assessment revealed a particular improvement t2b as well as an enhancement of the general image appearance (oai) when applying BM. These results were partially influenced by tumor grade and independent of the underlying model, i.e. also the more complex 2CXM resulted in substantially improved image quality.

DCE data are easily affected by a relatively low signal-to-noise ratio, and fitting the model to data results in noisy images that in severe cases impede reliable image interpretation. Different regularization techniques are usually employed to minimize extensive image noise [27, 28]. LM is a robust method, used successfully in many curve-fitting problems. The multi-dimensional non-linear deconvolution problem, inherent to the ETM and 2CXM models may, however, be afflicted by a too complicated cost-function surface to allow optimization by LM. Computer simulations have shown that especially v_e_ in the 2CXM gives rise to spurious high-intensity voxels unrelated to physiology (Robust estimation of hemo-dynamic parameters in traditional DCE-MRI models; submitted to PLoS One, PONE-D-18-01462). These voxels might be misinterpreted as representing regions with high and low perfusion values, respectively, or might obscure important, pathology-related structures. Quantitative studies are particularly affected by the inclusion of noisy voxels, as this will inevitably lead to non-physiological values in the resulting parameters. It was found that insufficient sampling time, in conjunction with slow extra-vascular tracer kinetics, resulted in ever-increasing concentration-time curves during the entire scan duration. Such effects were found to result in particularly poor estimations of LM-based v_e_ values, with both over- and underestimation occurring. Conversely, the BM approach was found to consistently underestimate v_e_, which results in smoother images. The noisy 2CXM-based v_e_ maps, encountered in the present work, are likely to be an extension of these findings. In brain tissue, and especially healthy white matter, relatively low blood volume induces only limited signal intensity variation. In such a case, the poor signal-to-noise ratio, affecting particularly the tail of the concentration curves, prevents the LM approach from producing meaningful v_e_ values. The type of curve-fitting algorithm appears to influence the appearance of parameter maps, which are, primarily, based on the first part of the contrast bolus passage to a much less extent. Very similar oai scores on e.g. F_p_ maps support this supposition.

BM differs from LM in various ways. It is, for instance, based on the maximization of a negative log-likelihood function, which we speculate represents a beneficial transformation of the optimization landscape. Recent clinical and phantom studies have indicated that BM might be more suitable than LM for perfusion data [15–18, 20, 29, 30]. Our results are therefore in line with these findings, showing the superiority of the Bayesian approach when generating hemodynamic and in particular leakage-related parameter maps.

We note that other research groups have previously published work on Bayesian methodology in the context of DCE hemodynamic parameter estimation [19, 20], which are similar the one presented in this and the associated work (“Robust estimation of hemo-dynamic parameters in traditional DCE-MRI models”; submitted to PLoS One, PONE-D-18-01462). There are, however, some significant differences. First, the algorithm used here utilizes the measured arterial input functions rather than, which is quite common in DCE studies, a bi-exponential fit to a measured input function or a universal input function [31]. Second, the delay between the site of measurement of the AIF and any particular tissue curve is an adaptive parameter in our model alongside the actual hemodynamic parameters, such as F_p_ and v_e_.

We have demonstrated the superiority of BM in glioma, which are mostly solitary brain lesions. We note, however, that spurious voxels on leakage-related LM-based parameter maps were most pronounced in normal appearing tissue on leakage-related LM-based parameter maps, and they might therefore prevent the detection of peri-lesional changes or small satellite tumors. One might argue that spurious voxels have less impact on the diagnostic performance of DCE. We showed, however, that tumor grades could only be differentiated reliably on BM-based, but not on LM-based v_p_ maps. This was not the case for leakage-related parameter maps. 40% of our low-grade gliomas showed contrast enhancement in parts of the lesion, explaining the reduced performance of K_1_, regardless of the underlying fitting algorithm. This was, however, the case for ETM-based K_trans_, which is known to be considerably influenced by blood flow, a strong discriminator of tumor grades.

A limitation to the application of curve fitting approaches in a clinical setting is the relatively high demand on computational resources, which implies long post-processing times. Processing time can, however, be sped up considerably by grouping of voxels with similar signal curve characteristics or through the use of parallel processing paradigms, such as multiple compute threads, distributed computing, and using graphics processing units for computationally intensive tasks.

Another technical limitation pertains to the assumption that the functional form of the AIF represents the actual input supplied to each voxel. Hence, dispersion effects, local T1 effects, and other phenomena are not handled adequately, which may result in incorrect hemodynamic parameters. This is discussed in greater detail in “Robust estimation of hemodynamic parameters in traditional DCE-MRI models” (submitted to PLoS One, PONE-D-18-01462). While potentially representing a general issue, we have in this work chosen to allow the Bayesian model to adapt to the curve without posing restrictions on the parameters. Taking the simple ETM as an example, this may in the case of v_e_ result in voxels with > 1 ml/100 ml. Imposing limits on that particular parameter would have resulted in hindered F_p_ determination, as F_p_ would have to compensate for these effects alone.

A limitation of our study is that only two readers rated the parameter maps. Both are experienced neuroradiologists, but with different degrees of expert knowledge in reading DCE-images, allowing the level of experience as an additional confounder. A further limitation is the natural heterogeneity of glioma. We included both low- and high-grade glioma, as our intention was to evaluate a typical clinical spectrum. Low-grade glioma can, however, be challenging to distinguish on all parameter maps, which is likely to influence the quality scores to some extent. This could, however, be argued to represent a strength, rather than a weakness of our study, as the method appears to be applicable to both glioma grades and thereby maybe also to other brain pathologies.

Out of ethical reasons, we refrained from performing DCE-guided biopsies and are therefore not able to debilitate possible satellite tumors outside the primary lesion or even in the contralateral hemisphere. Thorough evaluation of the conventional images makes it, however, unlikely that the numerous spurious voxels, particularly on LM-based leakage maps, represent dissemination.

In summary, we have demonstrated that BM has the potential to improve the quality of DCE-derived parameter maps in glioma patients, thereby increasing the diagnostic reliability of especially leakage-related images. Our DCE data could even be fitted to a more complex compartmental model, allowing the separation of flow and permeability, without compromising image quality. Our results suggest that integrating the Bayesian approach in clinical software solutions might increase the confidence and applicability of DCE imaging, in particular when using this technique in quantitative imaging studies, for example to evaluate new treatments affecting the blood-brain barrier. It is, moreover, possible that the assessment of more global brain diseases as multiple sclerosis, cerebrovascular diseases, dementia etc. that are characterized by subtle and widespread changes of the blood-brain barrier [32] may in particular profit of the BM approach.
